# Large Area Patterning of Highly Reproducible and Sensitive SERS Sensors Based on 10-nm Annular Gap Arrays

**DOI:** 10.3390/nano12213842

**Published:** 2022-10-31

**Authors:** Sihai Luo, Andrea Mancini, Enkui Lian, Wenqi Xu, Rodrigo Berté, Yi Li

**Affiliations:** 1 Department of Chemistry, Norwegian University of Science and Technology (NTNU), 7491 Trondheim, Norway; 2 Chair in Hybrid Nanosystems, Nanoinstitute Munich, Faculty of Physics, Ludwig-Maximilians-Universität München, Königinstrasse 10, 80539 München, Germany; 3 Department of Chemical Engineering, Norwegian University of Science and Technology (NTNU), 7491 Trondheim, Norway; 4 School of Microelectronics, MOE Engineering Research Center of Integrated Circuits for Next Generation Communications, Southern University of Science and Technology, Shenzhen 518055, China

**Keywords:** nanogaps, adhesion lithography, nanospheres lithography, surface-enhanced Raman spectroscopy, glucose, sensors

## Abstract

Applicable surface-enhanced Raman scattering (SERS) active substrates typically require low-cost patterning methodology, high reproducibility, and a high enhancement factor (EF) over a large area. However, the lack of reproducible, reliable fabrication for large area SERS substrates in a low-cost manner remains a challenge. Here, a patterning method based on nanosphere lithography and adhesion lithography is reported that allows massively parallel fabrication of 10-nm annular gap arrays on large areas. The arrays exhibit excellent reproducibility and high SERS performance, with an EF of up to 10^7^. An effective wearable SERS contact lens for glucose detection is further demonstrated. The technique described here extends the range of SERS-active substrates that can be fabricated over large areas, and holds exciting potential for SERS-based chemical and biomedical detection.

## 1. Introduction

Surface-enhanced Raman spectroscopy (SERS) has been widely used as a powerful tool capable of detecting molecules, biomedical sensing, and catalysis [1,2,3,4,5,6,7,8]. SERS substrates are crucial for SERS detection, due to the weak Raman signals of absorbed molecules being dramatically enhanced through tremendous electromagnetic hot-spots that are confined into nanoscale gaps or localized around sharp corners and edges of coinaged metal surfaces [9,10,11,12]. One remaining challenge in diverse applications of SERS lies in the fabrication of efficient SERS substrates that feature strong reproducibility and enhancement factors over large areas. Of particular interest is the creation of uniformly narrow gaps, with precise control of gap size on noble metal substrates, ideally using low-cost, high-yield, and time-saving techniques. Various nanofabrication methods, such as electron-beam/extreme-ultraviolet lithography (EBL/EUV) [13,14,15], focused-ion beam (FIB) milling [16,17,18], metal nanoparticles assembly [19,20,21], nanosphere lithography (NSL) [22,23,24], capillary force-assisted lithography [25,26], block copolymer lithography (BCL) [27,28], interference lithography [29,30,31], and DNA origami [32,33,34,35] have been developed and used to pattern highly hierarchical ordered metallic nanostructures with nanogap features for the purpose of making SERS substrates. Among these methods, for instance, EBL/EUV methods are still not stable and reproducible enough to create nanogaps down to 10 nm, which are well known for obtaining ultra-strong electromagnetic field enhancement [12,36], and, additionally, are cost-intensive and time-consuming for fabricating nanostructures over wide areas. Although some techniques, including FIB, metal nanoparticles assembly, and DNA origami, can fabricate very narrow gaps, they are either not applicable for low-cost, reproducibility, or high yield [37]. Therefore, a rapid, inexpensive, and reproducible strategy for patterning nanostructures with (sub-) 10 nm gaps over large areas is required.

Adhesion lithography, a new patterning approach based on thin-film deposition, molecular self-assembly, and peeling-off, can create gaps of a few nanometers or less [38,39,40,41]. In this work, we report a cost-effective approach for reproducible and high-throughput parallel fabrication of annular gap arrays (AGAs) with 10 nm gap size by combining nanosphere lithography and adhesion lithography. We demonstrate that these reproducible AGAs, over a centermeter^2^ area, exhibit excellent SERS performance for rhodamine 6g (R6G) detection at a concentration as low as 10^−12^ M. This substrate is recyclable for repeated SERS measurements simply by plasma cleaning. In addition, the AGAs are also used as SERS contact lenses for the detection of glucose in tears. Finite difference time domain (FDTD) simulations further indicate that well-defined 10-nm annular gaps are the main contributors to the electric field, underpinning the observed SERS performance.

## 2. Results and Discussion

**Figure 1:**
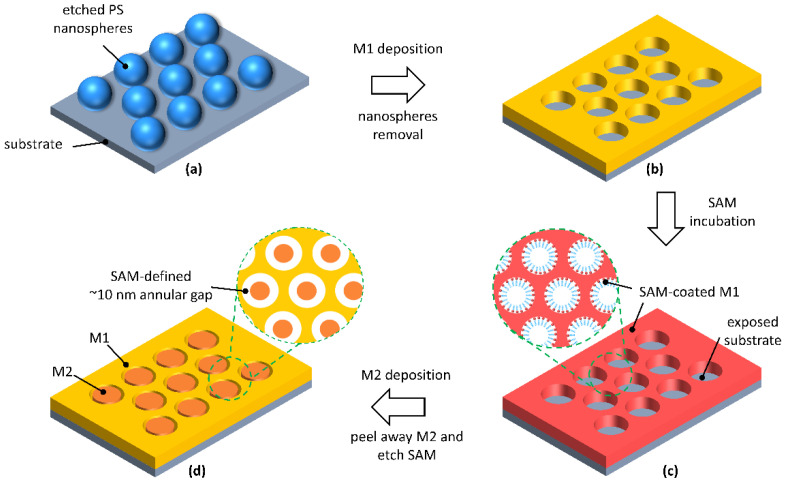
A schematic illustration of the fabrication process for AGAs. First, close-packed polystyrene nanospheres were self-assembled on a glass substrate, then oxygen plasma was used to etch nanospheres to the desired diameter (**a**). A thin metal (M1) film was deposited over the etched nanospheres, which are then stripped away by tape, leaving nanohole arrays (**b**). Then, a selected SAM is used to attach conformally to the nanohole arrays surface via solution–phase deposition (**c**). A second metal (M2) film is then deposited over the entire substrate. An adhesive material is applied on the top of M2 and then selectively peeled off, creating two metals, M1 and M2, sitting in a complementary arrangement side-by-side, separated by the SAM. Simple removal of the SAM using oxygen plasma reveals M1/air/M2 annular gaps with a nanoscale separation determined by the length of the selected SAM (**d**).

**Figure 2:**
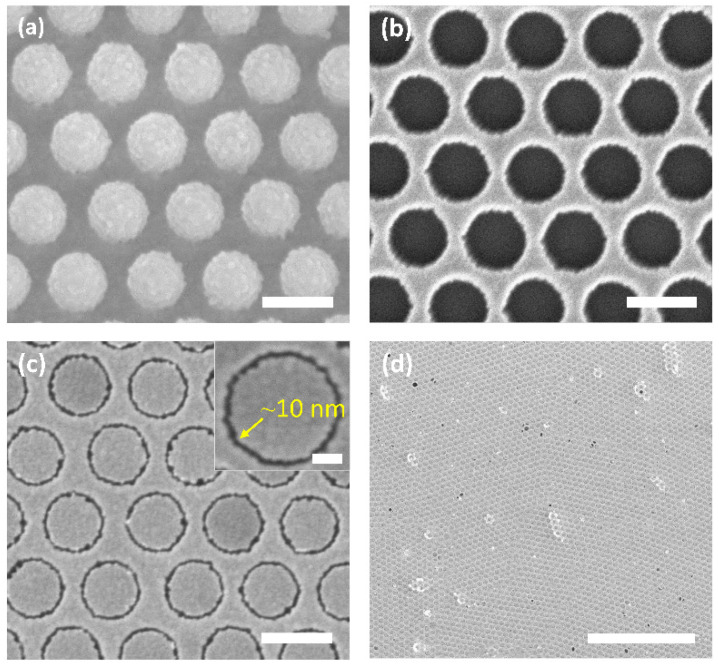
(**a**–**c**) Scanning electron micrographs (SEMs) of the corresponding processing steps in Figure 1 to fabricate AGAs, in which M1 and M2 are both gold. Scale bar: 500 nm and 50 nm (Inset in c) (**d**) Patterned gold AGAs over a large area, scale bar: 10 µm.

**Figure 3:**
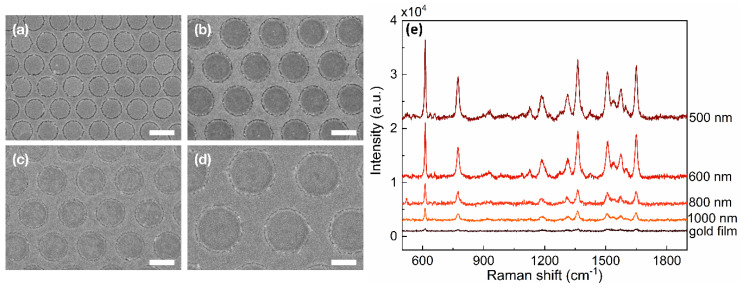
SEMs of gold AGAs with different periodicities (**a**–**d**): AGAs-P500, AGAs-P600, AGAs-P800, and AGAs-P1000. Scale bar: 500 nm. (**e**) Associated Raman spectra of 10^−4^ M R6G on these gold AGAs substrates and flat gold substrate.

The fabrication process for making the AGAs substrate is depicted in Figure 1. First, polystyrene (PS) nanospheres were assembled on a pre-cleaned glass substrate, which had their diameter reduced by oxygen plasma etching (Figure 1a). Then, a thin metal film (M1) was deposited over the nanospheres and the etched nanospheres were removed by tape-stripping, leaving M1 nanohole arrays (Figure 1b). An methyl-terminated metallophilic self-assembled monolayer (SAM) was conformally attached on the top and vertical sidewall surfaces of M1 nanoholes via solution-phase deposition (Figure 1c), followed by deposition of a second metal film (M2) [38,41]. For the formation of the annular nanogap arrays, an adhesive material was applied uniformly to the surface of M2, and then selectively peeled away, creating two metals sitting in a complementary arrangement side-by-side, spaced by the SAM. Simply removing the SAM using oxygen plasma cleaning reveals M1-air-M2 nanoring gaps, as shown in the Figure 1d. Figure 2 shows the scanning electron micrographs (SEM) of the corresponding processing steps for gold AGAs, using octadecanethiol (ODT) as SAM. The structural parameters are as follows: periodicity *P* = 500 nm, inner radius *r* = 180 nm, outer radius *D* = 190 nm, and the thickness of gold film is 60 nm. As shown in Figure 2c, well-defined annular gaps were formed along the pre-nanospheres-templated nanohole edges, where the gap size was defined by the length of the SAM—a few nanometers or less—which, in the resultant structures, is about 10 nm (Figure S1, Atomic Force Microscopy image). Notably, the dense AGAs can be parallel fabricated over centermeter^2^-sized areas, which is not feasible by conventional lithography techniques, such as EBL/FIB. Figure 3a–d show the as-fabricated gold AGAs substrates with varied periodicities ranging from 500 nm to 1000 nm (AGAs-P500, P600, P800 and P1000), and the corresponding close-up images for each periodicity are shown in Figure S2. Additionally, the size of a single nanoring, which is pre-defined by the diameter of templated PS spheres, can be also tuned directly by changing etching time of the oxygen plasmon process (see Figure S3). Moreover, different metal annular gap arrays, such as silver/silver, aluminum/aluminum, and gold/silver AGAs, were also fabricated using the above procedures, respectively (see Figure S4). Therefore, such AGAs fabricated by our method provide increased design freedom in engineering SERS substrates and extending applications of AGAs [42,43,44].

Such nanoscale ring cavities can generate tremendous electromagnetic field enhancements, as well as localization of incident light, and thus are suitable for SERS analysis. The AGAs substrates made of gold were selected due to their inert chemical nature. Next, R6G, a typical Raman probe molecule, was applied as an example to investigate the SERS activities of gold AGAs substrates. As shown in Figure 3e, a negligible Raman signal is observed on a flat gold substrate due to the low concentration of R6G (10^−4^ M), while gold AGAs substrates exhibit highly enhanced Raman signals of R6G, which can be mainly ascribed to the presence of annular gaps, and, thus, the generation of intense hot spots [45]. Notably, the Raman intensity decreases, as expected, as the periodicity increases, which is in line with previous reports [46], indicating that gold AGAs-P500 has a high SERS activity.

**Figure 4:**
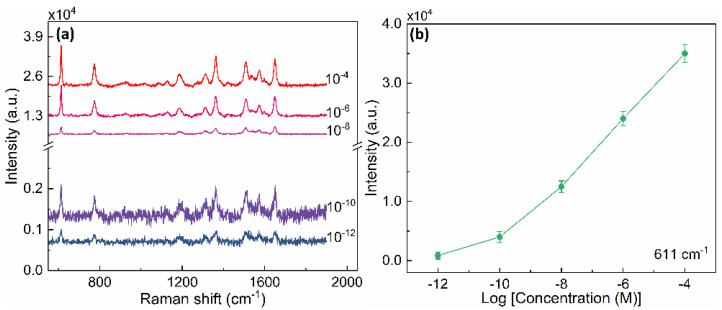
(**a**) Raman spectra of R6G molecules on gold AGAs-P500 with different concentrations, ranging from 10^−4^ M to 10^−12^ M. A scale break is deliberately inserted in the vertical axis in order to visualize weak Raman peaks. (**b**) The relationship between the Raman intensity at a peak of 611 cm^−1^ of R6G molecules and concentrations.

**Figure 5:**
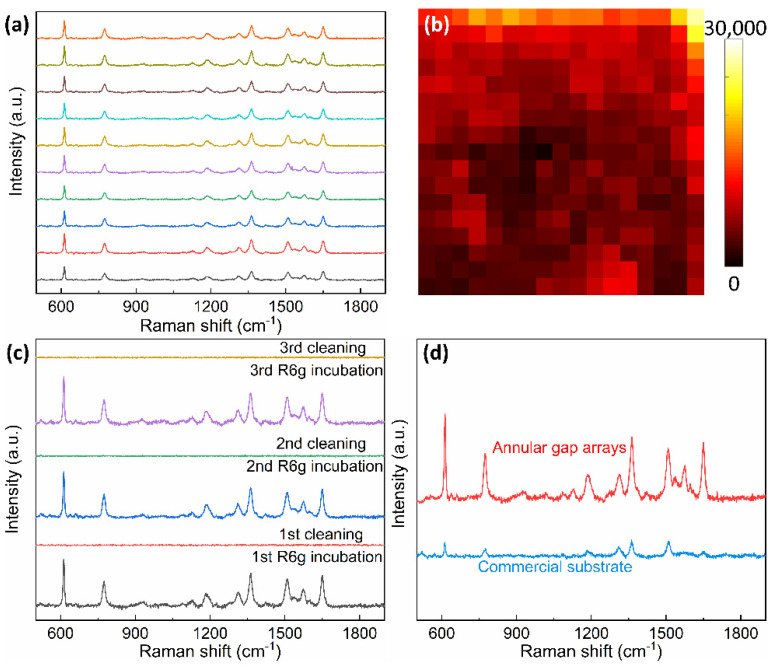
(**a**) Randomly collected ten Raman spectra of R6G molecules at a concentration of 10^−4^ M over 1 mm^2^ on gold AGAs-P500. (**b**) Raman mapping image of 10^−4^ M R6G on the gold AGAs substrate over 17 µm × 17 µm with a step size of 1 µm. (**c**) Measured Raman spectra from the same gold AGAs-P500 substrate after 3 cycles of R6G incubating and oxygen plasma cleaning. (**d**) Raman spectra of R6G molecules at concentration of 10^−4^ M on the gold AGAs-P500 substrate and the commercial Hamamatsu gold SERS substrate.

The sensitivity of the gold AGAs-P500 was further investigated by varying the concentration of R6G from 10^−4^ to 10^−12^ M. The Raman intensity decreases gradually with decreasing concentration (Figure 4a,b). When the concentration of R6G is as low as 10^−12^ M, the characteristic peaks of R6G can be still observed, which reveals the excellent Raman sensitivity of the gold AGAs-P500 as an efficient SERS platform. Furthermore, as presented in the Figure 5a, ten Raman spectra from randomly collected Raman signals on ten spots of the substrate over 1 mm^2^ are exceptionally reproducible and stable, which indicates high spot-to-spot reproducibility and reliability of the gold AGAs-P500 substrate. Figure 5b shows the Raman mapping of the 10^−4^ M R6G on AGAs-P500 substrate over 17 × 17 µm^2^, indicating the outstanding homogeneity of SERS intensity. In addition, the gold AGAs-P500 substrate also exhibits excellent reusability as a SERS substrate. It allows multiple cycles of SERS measurements on the same substrate after cleaning away the previous analytes via oxygen plasma etching, which benefits from the vertically aligned annular gaps on the substrate. Figure 5c shows that the substrate can be used for repeated SERS measurements after 5 min of oxygen plasma cleaning, where three cycles of R6G incubation and plasma cleaning are presented. The characteristic peaks of R6G disappeared after every cycle of plasma cleaning, and no other peaks were observed, suggesting that the byproducts generated during the oxygen plasma process are weakly adsorbed. This showed the excellent reusability of the substrate for SERS application. Figure 5d shows the comparison between the gold AGAs-P500 substrate with active area of over 1 cm^2^, and the commercial Hamamatsu SERS substrate with active area of 0.8 cm^2^. It is clear that gold AGAs-P500 exhibits exceptional SERS ability for detecting R6G with a concentration of 10^−4^ M, which provides a promising alternative platform for practical SERS applications. As a result, the excellent SERS sensitivity and reproducibility of the gold AGAs-P500 substrate could be attributed to the following reasons: (a) surface plasmon resonance excited by incident light in the substrate dominantly contributes to the significant enhancements and localizations of the electric field at the nanoring gaps (hotspots); (b) the easy access and homogeneous adsorption of analytes that benefit from the uniform vertically aligned AGAs on the substrate; (c) the large area periodic nanostructures offer excellent reproducibility of SERS signals. The averaged enhancement factor (EF) of the gold AGAs-P500 substrate is of near 10^8^ (~7.2 × 10^7^, see EF calculation in supporting information), which is comparable to some recently reported SERS substrates in terms of detection of R6G and large area fabrication (Table S1), indicating that the gold AGAs-P500 substrate has a high SERS activity.

**Figure 6:**
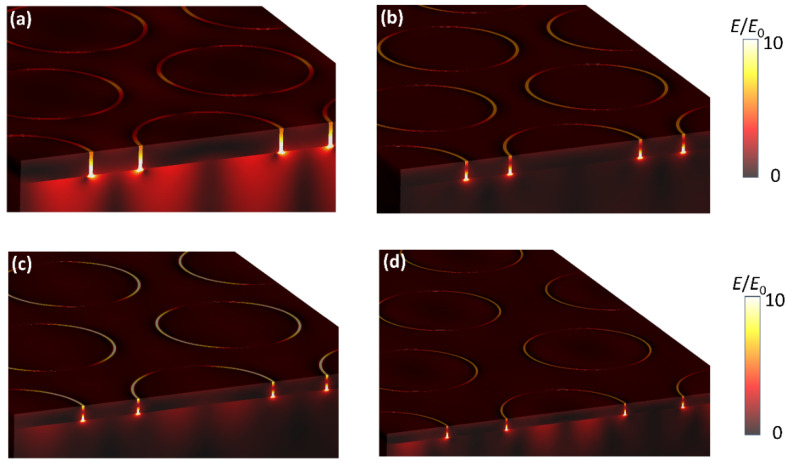
3D FDTD simulations of near-field electric field distributions of AGAs-P500 (**a**), AGAs-P600 (**b**), AGAs-P800 (**c**), and AGAs-P1000 (**d**) under an excitation wavelength of 633 nm.

In order to elucidate the electric field distribution of gold AGAs substrates, a 3D finite difference time domain (FDTD) simulation was performed. Figure 6 illustrates 3D electric field intensity distributions of the corresponding AGAs substrates, as shown in the Figure 3, at an excitation wavelength of 633 nm. It is evident that electric fields are all highly intense compared with the incident field and strongly localized at nanoring gap regions, and that the localized electric fields maxima are near the bottom of nanogap, due to the high dielectric of the substrate. Apparently, gold AGAs-P500 substrates demonstrate higher electric field intensity than that of AGAs-P600, AGAs-P800, and AGAs-P1000 substrates, which agrees well with the experimental results.

**Figure 7:**
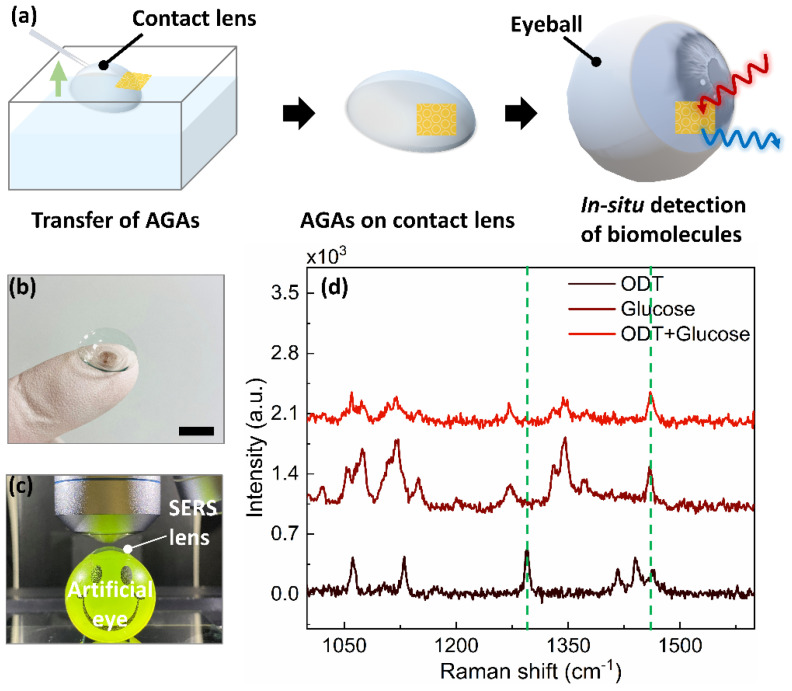
Fabrication of SERS contact lens and demonstration of glucose detection. (**a**) Schematic illustration of the fabrication of SERS contact lens via wet etching transfer process. (**b**) Optical images of hydrogel lens with transferred gold AGAs-P500. Scale: 5 mm. (**c**) Optical image of envisaged setup for detecting glucose SERS signal. (**d**) Raman spectra of glucose with a concentration of 1 mM, showing the successful detection of glucose.

Diabetes resulting from a high level of glucose is one of the metabolic diseases that endanger human health. The level of glucose is, therefore, of great significance for the diagnosis, monitoring, and treatment of clinical diabetes [47]. Although a high fasting glucose level in blood can be diagnosed as a sign of diabetes, due to the inconvenience and infection risk of blood tests, non-invasive and in situ monitoring of glucose levels has been extensively attempted [48,49]. Considering that glucose is present not only in blood, but also in tears [50], the accurate monitoring of the glucose level in tears by employing smart sensors, such as a contact-lens-type sensor, is expected to be an alternative approach for non-invasive glucose monitoring. We demonstrated the versatility of our method for unconventional application of SERS by employing a “SERS contact lens,” which can potentially enable detection of glucose levels in tears. The SERS lens can be simply fabricated by transferring gold AGAs onto commercially available contact lenses, as presented in Figure 7a. The fabricated SERS lens (gold AGAs-P500) was incubated in a 1 mM ethanol solution of ODT in order to form a uniform monolayer, which could act as a partition layer during glucose analysis [51]. Figure 7b shows a photograph of the fabricated SERS lens on a commercially contact lens. A drop of 1 mM (which corresponds to the glucose level in the tears of diabetes patients) aqueous solution of glucose was released onto the fabricated SERS lens, which was mounted on an artificial eye (Figure 7c). Figure 7d shows the obtained Raman spectra, in which the characteristic Raman peaks at 1270 cm^−1^, and 1461 cm^−1^ of glucose molecules were clearly detected with a detection limit of 0.1 mM (Figure S5). However, for the real practical applications of the SERS contact lens, the applicability of retina-safe laser excitation also remains a huge challenge [47,52]. These need to be properly addressed for further development of future non-invasive optical wearable devices.

## 3. Methods

### 3.1. Materials

In all experiments, deionized water (18.2 MΩ·cm) was obtained from a Millipore filtration system (Milli-Q IQ7003, Darmstadt, Germany). The silicon wafer (p-doped) or borosilicate glass substrates were cleaned by oxygen plasma before use. The self-assembled molecules of Octadecanethiol (ODT, 98%), PS spheres aqueous suspension (10 wt%), Rhodamine 6G (R6G, 99%), and glucose (99.5%) were all purchased from Sigma Aldrich (Trondheim, Norway). In addition, acetone and absolute ethanol (99.5%, VWR chemicals, Trondheim, Norway) were used as received without further purification.

### 3.2. Fabrication of Gold AGAs Substrates

A piece of glass or silicon wafer was cleaned with acetone, ethanol, and deionized water, then dried by N_2_ stream followed by oxygen plasma for 3 min. Then, a 0.4 µL droplet of polystyrene (PS) nanospheres diluted in 2:1 (*v/v*) Milli-Q water: ethanol was placed onto the glass within a 1 cm diameter PDMS-defined well, and dried at ambient condition. The close-packed nanospheres were then etched with oxygen plasma (power 100 W, O_2_ flow rate 50 sccm), causing them to shrink while retaining their original location. Afterwards, a 60 nm-thick gold layer (5 nm Titanium as adhesion layer) (M1) was deposited onto the templated substrate by e-beam evaporation at 2 Å/s under 5 × 10^−7^ Torr. Then, the PS nanospheres were removed by 3M scotch tape, leaving behind the gold nanohole arrays. The substrate was then cleaned by oxygen plasma for 3 min, before being soaked in a 2 mmol ethanol solution of ODT for 2 h. The ODT-coated substrate was then thermally annealed at 80 °C for 30 s in air, before rinsing lightly in ethanol to remove unbound/residual ODT molecules. A second 60 nm gold layer (M2) was deposited over the entire substrate. Finally, the M2 layer was partially removed by gently applying an adhesive material and peeling it off, forming the SAM-defined gold AGAs [38,41]. The SAM was removed by oxygen plasma for 5 min (power 100 W, O_2_ flow rate 50 sccm).

The morphologies of the as-fabricated substrates were characterized by SEM on a FEI APREO scanning electron microscope (Hillsboro, OR, USA) at 5 kV and 13 pA. In order to fabricate SERS contact lenses, hydrofluoric (HF) acid was used to corrode the glass substrate in order to detach the gold AGAs-P500. The detached gold AGAs floated on the HF solution/air interface, and were then scooped up with clean glass substrates and released to the surface of pure deionized water (repeated 3 times to remove any residual HF). Finally, the floating AGAs were lifted up by a clean commercial contact lens.

### 3.3. Raman Measurements

Raman spectra were obtained on a Horiba confocal Raman spectrometer with laser excitation wavelength at 633 nm. The laser beam was focused onto the sample through a ×50 objective lens with selected 0.5 mW of laser power, for 10 s of acquisition time. In order to prepare the samples for SERS measurement, the samples were first immersed in the ethanol solution of R6G with different concentrations, ranging from 10^−4^ to 10^−12^ M, for about 4 h. The samples were then rinsed extensively with ethanol and dried using an N_2_ stream before Raman measurements. For the detection of glucose, the transferred AGAs film was firstly immersed in 1 mM ODT ethanol solution for 12 h to form a uniform monolayer; then, a drop of 1 mM of glucose aqueous solution was released onto its surface before conducting Raman measurements.

### 3.4. FDTD Simulations

A commercial software, FDTD solutions (Lumerical Solutions, Inc., Vancouver, BC, Canada), was applied in order to calculate the transmission spectra and near field *E*-field distributions of the annular gap structures with the same diameters. A rectangular lattice, consisting of one nanoring in the center and four quartering nanoring gaps at the four corners, was used to simulate an infinite array of annular gap. A non-uniform mesh was used in the entire simulation domain with 2 nm in the nanogap region, and the lateral and vertical boundary conditions were periodic and perfectly matched layer boundary, respectively.

## 4. Conclusions

We demonstrated a rapid, cost-effective, and high-throughput patterning of hexagonal packed AGAs for ultrasensitive and reproducible SERS platforms. The method reported was based on the SAM to attach conformally to a self-assembled templated metal layer, and, thus, weakened adhesion to a subsequently deposited metal film, where the resultant gap was defined by the length of the selected SAM, which is typically below 10 nm. Such a nanoscale annular gap shows significant electromagnetic field confinement, which has also been verified by FDTD simulations, and boosts Raman enhancements by at least one order of magnitude with respect to flat gold. We have demonstrated that the AGAs strongly exhibited the SERS signal of R6G at a concentration as low as 10^−12^ M, with an EF of up to ×10^7^. Moreover, the reported method also enabled the fabrication of a SERS contact lens that can detect glucose in an aqueous solution that corresponds to the glucose levels in the tears of diabetes patients. These promising results suggest that our method is likely to be extended for various nanoscale sensing platforms where patterning of high-resolution nanostructures is necessary, and the application of conventional lithography approaches remains challenging.

## Data Availability

The data presented in this study are available on request from the corresponding author.

## Supplementary Materials

The following supporting information can be downloaded at: https://www.mdpi.com/article/10.3390/nano12213842/s1, Figure S1. Atomic force microscopy image (AFM) of nanoring gap arrays (NRG-10); Figure S2. SEMs of close-up annular gap for each periodicity (P). (a) *P* = 500 nm (b) *P* = 600 nm (c) *P* = 800 nm (d) *P* = 1000 nm. Scale: 200 nm; Figure S3. SEMs of close-up single annular gap from an AGA array with *P* = 500 nm, in which the diameter (D) of inner disk was reduced by changing etching time (t) of oxygen plasma process. (a) *D* = 440 nm, *t* = 5 min, (b) *D* = 380 nm, *t* = 10 min (c) *D* = 280 nm, *t* = 15 min (d) *D* = 200 nm, *t* = 20 min. Scale: 50 nm; Figure S4. The SEMs of annular gap arrays with gap size of 10-nm, made of different metals. (a) silver–air–silver, (b) gold–air–aluminum, and (c) aluminum–air–aluminum. Scale: 200 nm; Figure S5. Raman spectra of glucose with different concentrations on AGA arrays substrates, indicating detection limit of 10^−4^ M; Table S1. Comparisons between some recent works and this work. References [23,25,27,45,46,52,53,54,55,56] are cited in the Supplementary Materials.
