# Supplementary material for: Large Area Patterning of Highly Reproducible and Sensitive SERS Sensors Based on 10-nm Annular Gap Arrays
Source: Nanomaterials (Basel). 2022 Oct 31;12(21):3842. doi: 10.3390/nano12213842 (PMC9655199; doi:10.3390/nano12213842)
Supplement: Supplementary file 1 [file nanomaterials-12-03842-s001.zip › nanomaterials-1966108-supplementary.pdf]

nanomaterials

Supplementary Materials

Large Area Patterning of Highly Reproducible and Sensitive SERS Sensors Based on 10-nm Annular Gap Arrays

Sihai Luo [1,*], Andrea Mancini [2], Enkui Lian [1], Wenqi Xu [3], Rodrigo Berté [2] and Yi Li [2,4]

[1] Department of Chemistry, Norwegian University of Science and Technology (NTNU), 7491 Trondheim, Norway
[2] Chair in Hybrid Nanosystems, Nanoinstitute Munich, Faculty of Physics, Ludwig-Maximilians-Universität München, Königinstrasse 10, 80539 München, Germany
[3] Department of Chemical Engineering, Norwegian University of Science and Technology (NTNU), 7491 Trondheim, Norway
[4] School of Microelectronics, MOE Engineering Research Center of Integrated Circuits for Next Generation Communications, Southern University of Science and Technology, Shenzhen 518055, China
* Correspondence: sihai.luo@ntnu.no

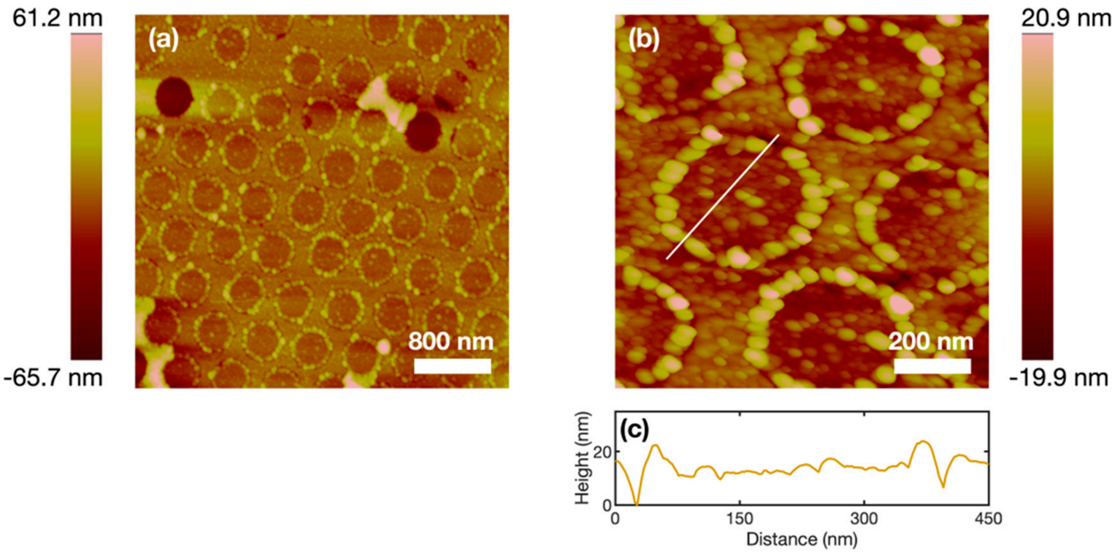

Figure S1. Atomic force microscopy image (AFM) of nanoring gap arrays (NRG-10).

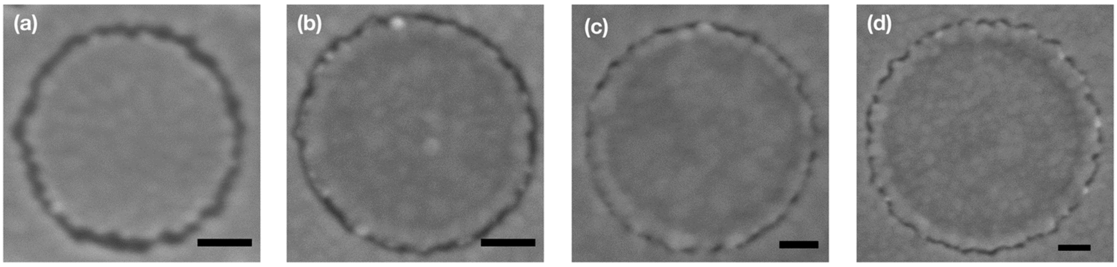

Figure S2. SEMs of close-up annular gap for each periodicity (*P*). (**a**) *P* = 500 nm (**b**) *P* = 600 nm (**c**) *P* = 800 nm (**d**) *P* = 1000 nm. Scale: 200 nm.

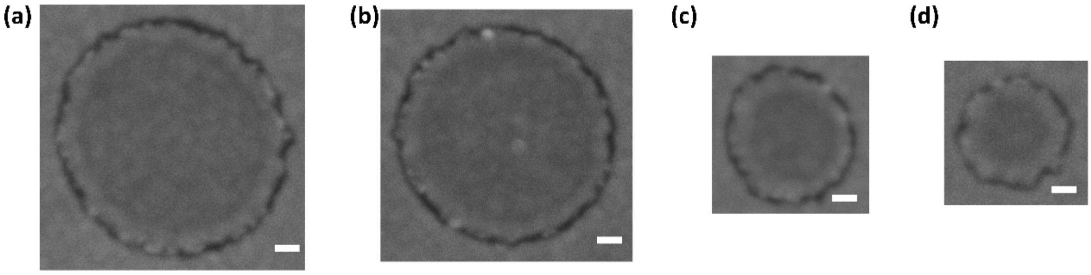

Figure S3. SEMs of close-up single annular gap from an AGA array with P = 500 nm, in which the diameter (D) of inner disk were reduced by changing etching time (t) of oxygen plasma process. (**a**) D = 440 nm, t = 5 min, (**b**) D = 380 nm, t = 10 min (**c**) D = 280 nm, t = 15 min (**d**) D = 200 nm, t = 20 min. Scale: 50 nm.

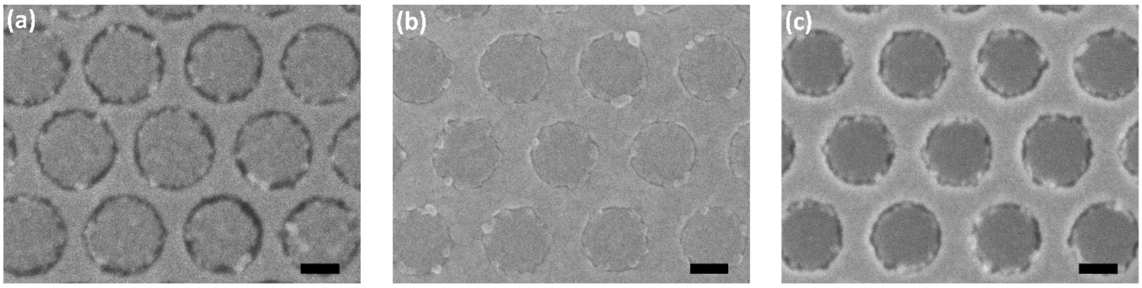

Figure S4. The SEMs of annular gap arrays with gap size of 10-nm, made of different metals. (**a**) silver-air-silver, (**b**) gold-air-aluminum and (**c**) aluminum-air-aluminum. Scale: 200 nm.

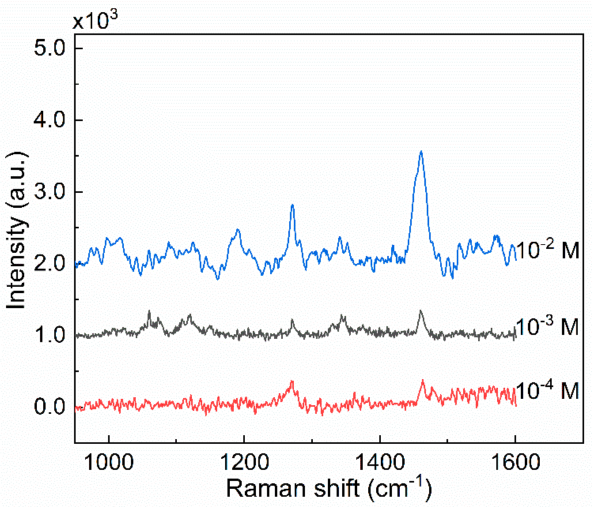

Figure S5. Raman spectra of glucose with different concentrations on AGA arrays substrates, indicating detection limit of 10^{-4} M.

Raman enhancement factor (EF) calculation

The EF of gold AGA arrays substrate was calculated using the Raman peak of 611 cm^{-1} of R6G according to the following equation [1]:

$$EF = \frac{I_{SERS}/c_{SERS}}{I_{NR}/c_{NR}} \quad (1)$$

Where I_{SERS} and I_{NR} are the Raman intensities of 10^{-12} M R6G on the AGA array substrate and 10^{-4} M R6G on the plain gold substrate as a normal Raman measurement reference, respectively, and c_{SERS} and c_{NR} are the concentrations of R6G involved in the SERS and normal Raman measurements. The I_{SERS} and I_{NR} are 1040.6 counts and 1449.7

counts, respectively. Thus, so Raman intensity ratio is estimated to be I_{SERS}/I_{NR} = 1040.6/1449.7 = 0.72. Therefore, taking all the parameters above into account, the EF can be estimated as following,

$$EF = \frac{I_{SERS}}{I_{bulk}} \times \frac{c_{SERS}}{c_{bulk}} = 0.72 \times \frac{10^{-4}}{10^{-12}} = 7.2 \times 10^{7} \quad (2)$$

Table S1. Comparisons between some recent works and this work.

Ref.	Technique	Structure	Characteristics
[2]	Atomic layer lithography, FIB milling	Ag nanoring cavity arrays	EF: 10^7<br>Detection limit: NA
[3]	Nanosphere lithography	Lotus seedpod-like structures	EF: 1.4 × 10^7<br>Detection limit: 10^{-12} M
[4]	Nanosphere lithography, chemical etching	Au nanoparticles functionalized Si nanorod arrays	EF: 3.3 × 10^7<br>Detection limit: 10^{-10} M
[5]	Sovlent-assisted nanotransfer printing	3D Cross-Point Plasmonic Nanoarchitectures	EF: 4.1 × 10^7<br>Detection limit: NA
[6]	Controlled evaporation deposition process	Vertically aligned Au nanorods	EF: NA<br>Detection limit: 10^{-15} M
[7]	Binary-template-assisted electrodeposition	Ag nanorod bundles	EF: 1.4 × 10^8<br>Detection limit: 10^{-9} M
[8]	Nanosphere lithography, reactive ion etching	Hierarchical Ag nanocone arrays	EF: NA<br>Detection limit: 10^{-17} M
[9]	Block copolymer lithography	Au-Ag Core-Shell Nanoparticle Arrays	EF: NA<br>Detection limit: 10^{-8} M
[10]	Solution-phase synthetic method	Au nanostar arrays	EF: 4.5 × 10^8<br>Detection limit: NA
This work	Nanosphere lithography, Adhesion lithography	Au annular arrays with sub-10-nm gap	EF: 7.2 × 10^7<br>Detection limit: 10^{-12} M

References

1. Le Ru, E.C.; Blackie, E.; Meyer, M.; Etchegoin, P.G. Surface Enhanced Raman Scattering Enhancement Factors: A Comprehensive Study. *J. Phys. Chem. C* **2007**, *111*, 13794–13803, https://doi.org/10.1021/jp0687908.
2. Im, H.; Bantz, K.C.; Lee, S.H.; Johnson, T.W.; Haynes, C.L.; Oh, S.-H. Self-Assembled Plasmonic Nanoring Cavity Arrays for SERS and LSPR Biosensing. *Adv. Mater.* **2013**, *25*, 2678–2685, https://doi.org/10.1002/adma.201204283.
3. Jin, B.; He, J.; Li, J.; Zhang, Y. Lotus Seedpod Inspired SERS Substrates: A Novel Platform Consisting of 3D Sub-10 nm Annular Hot Spots for Ultrasensitive SERS Detection. *Adv. Opt. Mater.* **2018**, *6*, https://doi.org/10.1002/adom.201800056.
4. Lin, D.; Wu, Z.; Li, S.; Zhao, W.; Ma, C.; Wang, J.; Jiang, Z.; Zhong, Z.; Zheng, Y.; Yang, X. Large-Area Au-Nanoparticle-Functionalized Si Nanorod Arrays for Spatially Uniform Surface-Enhanced Raman Spectroscopy. *ACS Nano* **2017**, *11*, 1478–1487, https://doi.org/10.1021/acsnano.6b06778.
5. Jeong, J.W.; Arnob, M.P.; Baek, K.-M.; Lee, S.Y.; Shih, W.-C.; Jung, Y.S. 3D Cross-Point Plasmonic Nanoarchitectures Containing Dense and Regular Hot Spots for Surface-Enhanced Raman Spectroscopy Analysis. *Adv. Mater.* **2016**, *28*, 8695–8704, https://doi.org/10.1002/adma.201602603.
6. Wei, W.; Wang, Y.; Ji, J.; Zuo, S.; Li, W.; Bai, F.; Fan, H. Fabrication of Large-Area Arrays of Vertically Aligned Gold Nanorods. *Nano Lett.* **2018**, *18*, 4467–4472, https://doi.org/10.1021/acs.nanolett.8b01584.
7. Zhu, C.; Meng, G.; Zheng, P.; Huang, Q.; Li, Z.; Hu, X.; Wang, X.; Huang, Z.; Li, F.; Wu, N. Silver-Nanorod Bundles: A Hierarchically Ordered Array of Silver-Nanorod Bundles for Surface-Enhanced Raman Scattering Detection of Phenolic Pollutants (Adv. Mater. 24/2016). *Adv. Mater.* **2016**, *28*, 4870–4870, https://doi.org/10.1002/adma.201670168.
8. Guan, Y.; Wang, Z.; Gu, P.; Wang, Y.; Zhang, W.; Zhang, G. An in situ SERS study of plasmonic nanochemistry based on bifunctional "hedgehog-like" arrays. *Nanoscale* **2019**, *11*, 9422–9428, https://doi.org/10.1039/c9nr01297d.
9. Cha, S.K.; Mun, J.H.; Chang, T.; Kim, S.Y.; Kim, J.Y.; Jin, H.M.; Lee, J.Y.; Shin, J.; Kim, K.H. Au–Ag Core–Shell Nanoparticle Array by Block Copolymer Lithography for Synergistic Broadband Plasmonic Properties. *ACS Nano* **2015**, *9*, 5536–5543, https://doi.org/10.1021/acsnano.5b01641.
10. Niu, W.; Chua, Y.A.A.; Zhang, W.; Huang, H.; Lu, X. Highly Symmetric Gold Nanostars: Crystallographic Control and Surface-Enhanced Raman Scattering Property. *J. Am. Chem. Soc.* **2015**, *137*, 10460–10463, https://doi.org/10.1021/jacs.5b05321.
